# A multifaceted intervention to improve syphilis screening and treatment in pregnant women in Kinshasa, Democratic Republic of the Congo and in Lusaka, Zambia: a cluster randomised controlled trial

**DOI:** 10.1016/S2214-109X(19)30075-0

**Published:** 2019-03-22

**Authors:** Fernando Althabe, Elwyn Chomba, Antoinette K Tshefu, Ernest Banda, María Belizán, Eduardo Bergel, Mabel Berrueta, Jane Bertrand, Carl Bose, Maria Luisa Cafferata, Waldemar A Carlo, Alvaro Ciganda, France Donnay, Ezequiel García Elorrio, Luz Gibbons, Karen Klein, Jerker Liljestrand, Paul D Lusamba, Arlette K Mavila, Agustina Mazzoni, Dalau M Nkamba, Friday H Mwanakalanga, Abigail Mwapule Tembo, Musaku Mwenechanya, Lee Pyne-Mercier, Cintia Spira, Jean D Wetshikoy, Xu Xiong, Pierre Buekens

**Affiliations:** a Institute for Clinical Effectiveness and Health Policy, Buenos Aires, Argentina; b University Teaching Hospital of Lusaka, Lusaka, Zambia; c Kinshasa School of Public Health, University of Kinshasa, Kinshasa, Democratic Republic of the Congo; d Tulane University School of Public Health and Tropical Medicine, Los Angeles, CA, USA; e University of North Carolina (UNC) School of Medicine, UNC Hospitals, Chapel Hill, NC, USA; f Department of Pediatrics, University of Alabama at Birmingham, Birmingham, AL, USA; g UNDP-UNFPA-UNICEF-WHO-World Bank Special Programme of Research, Development and Research Training in Human Reproduction, Department of Reproductive Health and Research, WHO, Geneva, Switzerland; h Bill & Melinda Gates Foundation, Seattle, WA, USA

## Abstract

**Background:**

Despite international recommendations, coverage of syphilis testing in pregnant women and treatment of those found seropositive remains limited in sub-Saharan Africa. We assessed whether combining the provision of supplies with a behavioural intervention was more effective than providing supplies only, to improve syphilis screening and treatment during antenatal care.

**Methods:**

In this 18-month, cluster randomised controlled trial, we randomly assigned (1:1) 26 urban antenatal care clinics in Kinshasa, Democratic Republic of the Congo, and Lusaka, Zambia, to receive a behavioural intervention (opinion leader selection, academic detailing visits, reminders, audits and feedback, and supportive supervision) plus supplies for syphilis testing and treatment (intervention group) or to receive supplies only (control group). The primary outcomes were proportion of pregnant women who had syphilis screening out of the total who attended the clinic; and the proportion of women who had treatment with benzathine benzylpenicillin out of those who tested positive for syphilis at their first antenatal care visit. This trial is registered at ClinicalTrials.gov, number NCT02353117.

**Findings:**

The 18-month study period was Feb 1, 2016, to July 14, 2017. 18 357 women were enrolled at the 13 intervention clinics and 17 679 women were enrolled at the 13 control clinics at their first antenatal care visit. Syphilis screening was done in a median of 99·9% (IQR 99·0–100·0) of women in the intervention clinics and 93·8% (85·0–98·9) in the control clinics (absolute difference 6·1% [95% CI 1·1–14·1]; p=0·00092). Syphilis treatment at the first visit was done in a median of 100% (IQR 99·7–100·0) of seropositive women in intervention clinics and 43·2% (2·6–83·2) of seropositive women in control clinics (absolute difference 56·8% [12·8–99·0]; p=0·0028).

**Interpretation:**

A behavioural intervention, together with the provision of supplies, can lead to more than 95% of women being screened and treated for syphilis. The sole provision of supplies is sufficient to reach such levels of screening coverage but is not sufficient to ensure high levels of treatment.

**Funding:**

Bill & Melinda Gates Foundation.

## Introduction

Mother-to-child transmission of syphilis is a preventable major public health concern, particularly in low-income and middle-income countries; untreated maternal syphilis is associated with stillbirths, perinatal deaths, prematurity, and congenital infections.^1^, ^2^ Despite global incremental improvements in screening and treatment, nearly one million pregnant women are infected with syphilis each year, and without treatment, an estimated 350 000 women will have adverse birth outcomes.^3^

To eliminate mother-to-child transmission of syphilis, WHO recommends that coverage of syphilis testing in pregnant women and treatment coverage of women positive for syphilis should each be at least 95%.^4^, ^5^ Despite these recommendations, progress in reducing maternal syphilis has been poor, especially in Africa, the region with the greatest incidence of mother-to-child syphilis transmission. In 2012, among women who were estimated to be likely to have active syphilis, only 3% in the Democratic Republic of the Congo and 24% in Zambia were treated.^3^

The primary challenge to improving screening and treatment of syphilis during pregnancy in low-resource settings is often assumed to be insufficient access to supplies. Although there is evidence supporting this assumption,^6^, ^7^ other implementation research suggests that additional interventions might be needed to overcome barriers related to health providers' behaviours regarding treatment and increase the likelihood that the supplies are used.^8^ To improve syphilis screening and treatment during pregnancy, we designed a multifaceted behavioural intervention based on previous research experience.^8^, ^9^, ^10^ We assessed whether combining the provision of supplies with a multifaceted behavioural intervention was more effective than providing supplies only to improve syphilis screening and treatment in seropositive pregnant women attending antenatal care clinics in Kinshasa (Democratic Republic of the Congo) and Lusaka (Zambia).

**Research in context**

**Evidence before this study**

We did searches in PubMed and the Cochrane Library for systematic reviews and randomised controlled trials (RCTs) assessing strategies to improve syphilis screening and treatment in pregnant women during antenatal care. We searched with the terms “syphilis screening”, “syphilis treatment”, and “pregnancy” for items published up to May 1, 2018, in any language. We found two systematic reviews that included two cluster RCTs. Both trials compared clusters using point-of-care screening, test results, and treatment on the same day against clusters using conventional laboratory testing. The proportion of women screened at first antenatal care visit were significantly higher with the point-of-care strategy (99·9%) than with the conventional laboratory strategy (79·6%; p<0·001) in the Munkhuu trial; the Myer trial did not report on screening as an outcome. Although proportions of women treated were marginally significantly higher with the point-of-care strategy (98·9%) than with the conventional strategy (89·6%; p=0·05) in the Munkhuu study, they were not significantly different in the Myer trial (64·1% with point-of-care strategy *vs* 68·6% with conventional strategy; odds ratio 0·82; 95% CI 0·57–1·17). Another cluster trial provided kits with supplies, including rapid treponemal tests, to improve screening practices during antenatal care in clinics in Mozambique, using a stepped-wedge design. The study showed an increase in syphilis screening from 65·7% to 95·5% (p<0·0001), as well as an increase in same-day treatment from 60·8% to 86·2% (p<0·024). We identified no published RCTs assessing the effect of behavioural interventions to health providers on syphilis screening and treatment in clinics provided with rapid treponemal tests.

**Added value of this study**

To our knowledge, this is the first randomised trial to assess the effect of a multifaceted behavioural intervention together with the provision of supplies for point-of-care syphilis screening and treatment in antenatal care clinics in sub-Saharan Africa.

**Implications of all the available evidence**

Audit and feedback, the use of reminders, academic detailing visits, and opinion leaders have been shown to be effective strategies targeting health-care providers to implement a variety of evidence-based health-care interventions. Our results showed that it is possible to screen and treat more than 95% of women at the first antenatal care visit, which is the suggested coverage proposed by WHO towards the elimination of mother-to-child transmission of syphilis. The sole provision of point-of-care tests can also achieve high levels of screening; however, it might not be enough to ensure treatment.

## Methods

### Study design and participants

This 18-month facility-based, two-arm, parallel cluster randomised implementation trial was done at 26 antenatal care clinics (16 in Kinshasa and ten in Lusaka). Clinics serving a mean of at least 300 new pregnant women per year, with antenatal care providers qualified to perform screening tests and administer injectable treatments, and with no active quality improvement programmes for syphilis detection and management were eligible. All women attending their first antenatal care visit at the participating clinics in both sites from Feb 1, 2016, to July 14, 2017, were eligible to participate. All participants gave written informed consent. A detailed description of the antenatal care providers is included elsewhere.^11^ Briefly, in both sites, antenatal care is provided by nurses, midwives, counsellors, and physicians.

The sites' ethics review committees and partner institutions approved the trial (appendix). All participating clinics provided written agreement to participate, and their authorities acted as clinical ethical guardians. All eligible women provided written consent for data collection purposes. The protocol was in accordance with the Ottawa Statement.^12^

### Randomisation and masking

After a 9-month period of baseline data collection,^13^ we assigned clinics to either an intervention group or a control group; the data centre at the Institute for Clinical Effectiveness and Health Policy (Buenos Aires, Argentina) assigned clinics to groups using a covariate-constrained randomisation procedure.^14^ This procedure ensured that intervention groups were balanced with respect to the number of women attending first antenatal care visits, the number of women attended per health provider at each clinic, the frequency of women screened for syphilis, the frequency of women positive for syphilis, the type of clinic, and the country.

The study statisticians (EBe and LG) did the randomisation using the covariate information from the baseline data collection period. Each clinic was informed of the randomisation allocation after baseline data collection and before implementation of the intervention. The nature of the trial precluded masking of randomisation allocation.

### Procedures

The intervention lasted for 18 months. Intervention strategies combined strategies shown to change providers' behaviour^8^, ^9^, ^10^ with those based on the diffusion of innovation theory,^15^ with the provision of supplies for syphilis screening and treatment. The intervention was tailored by formative research with clinic administrators, health providers, and pregnant women, which was done in Kinshasa and Lusaka and reported elsewhere.^16^ Briefly, the main barriers to immediate same-day treatment were unawareness that one dose of benzathine benzylpenicillin is enough to prevent mother-to-child syphilis transmission and concerns about potential adverse reactions to benzathine benzylpenicillin.^14^

The behavioural strategies used in the intervention group consisted of selection of opinion leaders, academic detailing visits with antenatal care providers, reminders, audits and feedback, supportive supervision, and the preparation of kits with the supplies for syphilis screening and treatment. Supplies provided to both groups were point-of-care rapid syphilis tests (Alere Determine Syphilis TP test; Alere International, UK) in Kinshasa and rapid immunochromatographic tests (SD Bioline Syphilis 3.0 test; Standard Diagnostic, Yongin, Korea) in Lusaka; treatment packs of benzathine benzylpenicillin 2·4 million international units (IU), syringe and needle, instructions and information on side-effects, and erythromycin 250 mg tablets; and anaphylaxis treatment for emergency use according to local practice guidelines. In accordance with WHO and country guidelines,^5^ all antenatal care providers were trained to administer one intramuscular dose of benzathine benzylpenicillin (2·4 million IU) to every woman with a positive syphilis test result at the first antenatal care visit to prevent mother-to-child syphilis transmission. Women who reported being allergic to penicillin were treated orally with 2 g per day of erythromycin for 14 days.

One or two antenatal care providers who were knowledgable, humane, and willing to share knowledge were identified at each intervention clinic with the use of a previously validated sociometric questionnaire and trained as facilitators for the study.^17^ These facilitators were trained by local coordinators in a 2-day workshop, which focused on how to screen for syphilis during pregnancy, training on the use of point-of-care tests, and how to treat and counsel pregnant women who were seropositive for syphilis. Additionally, facilitators were trained in monitoring the stock of supplies.

At the clinics, the facilitators, with the support of the intervention site coordinators, replicated the training for the rest of the antenatal care providers, and visited them individually to assess their views (academic detailing visits). Additionally, facilitators developed reminder materials for syphilis testing and treatment and management of anaphylaxis and placed them in waiting rooms and antenatal care offices. They also packaged the supplies in kits to ensure that they were readily available.

A local site coordinator did monthly supportive supervision activities with facilitators at each intervention clinic, which included monitoring intervention activities, implementation problem solving, and sharing and discussing a monthly report on the clinic's syphilis screening and treatment activities (audit and feedback). The facilitators then shared and discussed these reports with all antenatal care providers at the clinic. Monthly, the local site coordinator monitored the expiration dates and assessed the capacity of the antenatal care providers to correctly interpret the results of the point-of-care test in a random sample of providers during the visit by direct observation.

The intervention was implemented pragmatically in the context of routine antenatal care. First, the Kinshasa and Lusaka site coordination teams received intervention training at a local venue. Following each site's in-country central training, each site team developed a detailed implementation plan, identified facilitators, and then conducted their training at intervention clinics. At the clinics, the interventions started upon completion of the intervention training and provision of supplies. Details regarding the intervention in accordance with the template for intervention description and replication guide^18^ are described in the appendix.

Clinics in the control group continued routine antenatal care practice without a behavioural intervention but were provided with the supplies for syphilis testing and treatment and training in their use.

Outcome data were collected by trained study personnel at all participating clinics. Women were recruited consecutively. For all enrolled women, study personnel completed an antenatal care form with data regarding the practices and procedures performed during the first antenatal care visit, namely testing for syphilis, proteinuria, anaemia, and HIV and information about their obstetric and syphilis history. These data were obtained from the source documents that each clinic routinely uses to report antenatal care practices and procedures, including antenatal care log books, antenatal cards, prevention of mother-to-child transmission books, laboratory log books, and nursing log books. Data not available in source documents were collected by interviewing the women.

Data were collected on paper forms and entered in each country into a secure web-based, open-source data management system (OpenClinica), placed into a password-protected server, and securely transmitted using end-to-end encryption. The data entry system allowed range and consistency checks to be done. Cross-form edits were done at the data co-ordinating centre in Buenos Aires and resolved locally. We used double data entry to prevent data keying errors in all data forms.

Although the outcomes of the study were only measured at the first antenatal care visit, all women enrolled at participating clinics with a positive test for syphilis who did not receive treatment during the first visit were followed up by study personnel until their estimated date of delivery to ensure that all infected women received treatment before delivery. Women were contacted at the subsequent antenatal care visits or by phone. The list of seropositive women who did not receive treatment at the first visit was communicated monthly to the country principal investigators to be shared with local health authorities.

### Outcomes

The primary outcomes were the proportion of women screened for syphilis during their first antenatal care visit and the proportion of women with a positive syphilis test who were treated with benzathine benzylpenicillin (or erythromycin if applicable) during their first antenatal care visit. Secondary outcomes were the frequency of women screened for HIV, anaemia, and proteinuria at their first antenatal care visit, as other practices that could be affected by the behavioural intervention. We also did an analysis of the effects on primary outcomes by country; however, no statistical interaction was tested because the study was not designed to test such hypothesis.

### Statistical analysis

The statistical power was estimated for the two primary outcomes under the assumption that a mean of 300 women were initiating antenatal care per clinic each year and that three of them (1%) would be found seropositive at syphilis screening. A sample size of 20 clinics (ten per group) would provide more than 80% power to detect an increase in treatment of seropositive women at the first visit from 50% to 85%, with an α level of 0·025, and an intracluster correlation coefficient of 0·01. 20 clinics would also provide more than 90% power to detect an increase in the number of women screened for syphilis from 50% to 75%. Our calculations were based on the conservative assumption that 50% of women were screened and 50% of those found seropositive were treated at the participating clinics. To allow for clinics to drop out or to be excluded before group assignment, we collected baseline data from 29 clinics.

Analyses were performed according to the intention-to-treat principle, and no clinic was excluded from the analysis after allocation. All outcomes were analysed using the clinic as the unit of analysis. We calculated the proportion of all pregnant clinic attendees who were screened and the proportion of syphilis-positive women who were treated at each clinic for the 18-month follow-up period and compared the median proportions between the intervention and control clinics. The absolute difference between median outcome rates was calculated as the intervention effect and tested with the use of the Wilcoxon rank sum test with continuity correction to establish the 95% CI.^19^ Because a restricted randomisation procedure was used, a restricted randomisation test was also done.^20^ The results were similar to those obtained with the non-parametric strategy. All analyses were done with the R statistical package, version 1.1.456.

The syphilis screening outcome was the proportion of pregnant women who were screened for syphilis in each participating clinic at the first antenatal care visit out of all pregnant women attending a first visit. The treatment outcome was the proportion of women who were treated with one dose of benzathine benzylpenicillin during the first antenatal visit out of all women who tested seropositive at screening. Prevalence of syphilis infection was the number of women who were seropositive out of all those who were screened.

An independent data safety monitoring board reviewed the progress of the trial as specified in the protocol. The trial is registered at ClinicalTrials.gov, number NCT02353117.

### Role of the funding source

The funder of the study was involved in the study design and data interpretation and reviewed and approved the report, but had no role in data collection or data analysis. FA, MBer, MLC, KK, AC, EBe, and LG had access to all data in the study and the corresponding author had final responsibility for the decision to submit for publication.

## Results

**Figure 1:**
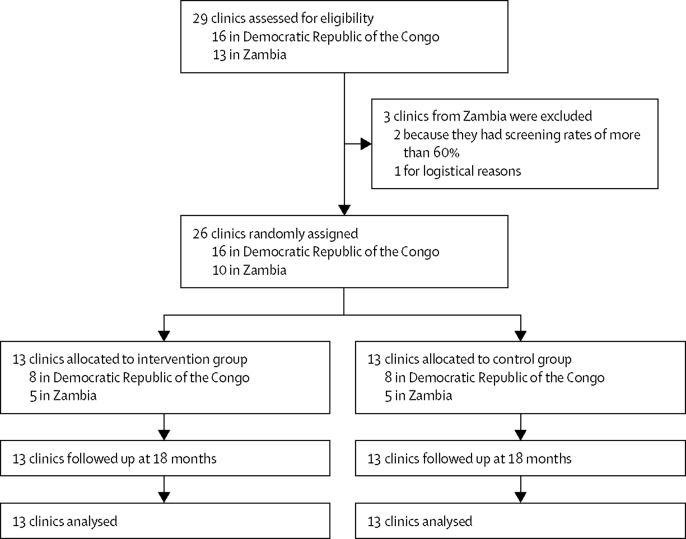
Trial profile

29 clinics completed baseline data collection, and three clinics in Zambia were excluded after the baseline data collection period, two of them because they had proportions of women tested for syphilis of more than 60% and one for logistical reasons. 26 clinics were randomly assigned to either the intervention (n=13) or control (n=13) group (figure 1). All 26 clinics completed the trial. Baseline data were collected for 9282 women in the supplies plus clinics and for 9265 women in the control group. The two groups were balanced with respect to baseline characteristics of the clinics and the women (table 1). The median proportions of women screened for syphilis were 41·4% (IQR 25·1–69·7) in the intervention clinics and 40·9% (15·5–74·2) in the control clinics. In women screened and found seropositive, the median proportions of women treated were 0% (0·0–0·0) in the intervention group and 0·0% (0·0–21·7) in the control group.

**Table 1:** Characteristics of antenatal care clinics and women at baseline

		**Intervention group**	**Control group**
Number of clinics		13	13
Number of women		9282	9265
Number of women per clinic		1412 (1461)	1360 (1450)
Country			
	Democratic Republic of the Congo	8 (62%)	8 (62%)
	Zambia	5 (38%)	5 (38%)
Type of clinic			
	Public	7 (54%)	8 (62%)
	Private	2 (15%)	1 (8%)
	Faith-based	4 (31%)	4 (31%)
Clinic with syphilis or HIV programme		12 (92%)	12 (92%)
Number of first antenatal care clinic visits per clinic		491 (194–894)	337 (192–1225)
Number of first antenatal care clinic visits per health provider		129 (65–192)	81 (64–153)
Screened for syphilis		41·4% (25·1–69·7)	40·9% (15·5–74·2)
Positive for syphilis		1·2% (0·2–1·6)	1·1% (0·0–2·5)
Treated for syphilis		0·0% (0·0–0·0)	0·0% (0·0–21·7)
Screened for anaemia		27·9% (5·0–54·0)	34·7% (4·0–53·5)
Screened for proteinuria		4·4% (0·2–10·7)	0·8% (0·0–3·7)
Screened for HIV		97·9% (91·2–98·2)	91·2% (82·8–97·6)
Maternal age <20 years		16·5% (12·4–19·0)	17·6% (15·6–20·8)
Incomplete primary school		13·6% (10·1–27·3)	22·4% (18·9–26·9)
Married or with partner		88·5% (86·2–91·2)	89·1% (84·2–90·6)
No previous pregnancies		21·4% (19·2–22·9)	25·9% (23·7–28·1)
Previous abortion		28·9% (14·7–30·6)	21·8% (12·6–29·4)
Previous preterm birth		2·6% (1·6–4·3)	3·1% (2·0–3·4)
Previous low birthweight baby		2·4% (0·7–5·1)	5·1% (1·9–7·0)
Previous babies with congenital anomalies		0·0% (0·0–0·3)	0·1% (0·0–1·1)
Previous syphilis infection		0·6% (0·0–1·8)	0·4% (0·0–1·0)
Gestational age at first visit ≤20 weeks		48·0% (37·6–52·5)	46·5% (42·6–53·6)

Data are N, mean (SD), n (%), or median (IQR). The clinic is the unit of analysis. Health provider is the antenatal health-care provider that works in each participating clinic attending the mothers for antenatal care during pregnancy.

Overall, intervention compliance was high. The behavioural components were implemented with a compliance rate between 88% and 100% of what was planned (appendix). Neither the intervention nor control clinics ran out of stock of supplies for screening and treatment during the 18-month period.

During the follow-up period, data were collected from 18 357 women in the intervention clinics and 17 679 in the control clinics. The maternal characteristics did not differ from the baseline period or between the two trial groups (appendix). No data on primary outcomes were missing in either group.

**Figure 2:**
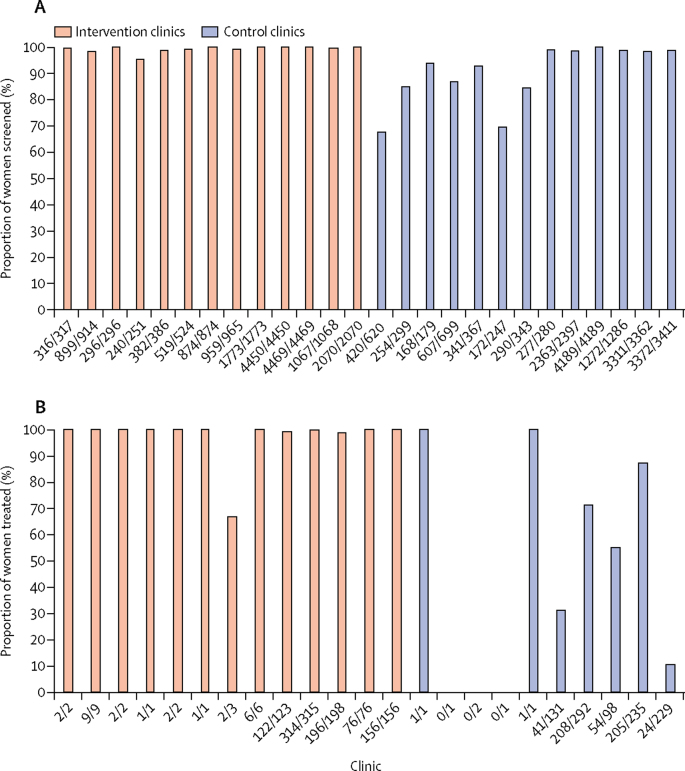
Syphilis screening and treatment across the clinics (A) Proportions of women screened at each of the 26 clinics. Numbers below bars are number of women screened over number of pregnant women attending clinic. (B) Proportions of women treated at the 23 clinics. Three clinics had no seropositive women, so proportion treated could not be calculated, and these clinics are not included on the graph. Numbers below bars are number of women treated over number of women who tested seropositive for syphilis.

Syphilis screening occurred in a median of 99·9% (IQR 99·0–100·0) of women attending the intervention clinics compared with 93·8% (85·0–98·9) attending the control clinics (table 2). The size of the intervention effect, measured as the median absolute difference between the two groups, was 6·1% (95% CI 1·1–14·1; p=0·00092). More than 95% of women were screened at all the intervention clinics (figure 2A). Of the 13 control clinics, six (46%) had screening proportions greater than 95% and seven (54%) had 68–94%. No substantial differences were noted between the observed effect in Kinshasa and Lusaka (appendix).

**Table 2:** Effect of the intervention on syphilis screening and treatment rates at the first antenatal care visit

	**Intervention group**		**Control group**		**Absolute difference between median proportions (95% CI)**	p value*
	Women (n=18 357), n/N	Clinics (n=13), median proportion† (IQR)	Women (n=17 679), n/N	Clinics (n=13), median proportion† (IQR)		
**Primary outcomes**						
Women screened for syphilis	18 314/18 357	99·9% (99·0 to 100·0)	17 036/17 679	93·8% (85·0 to 98·9)	6·1% (1·1 to 14·1)	0·00092
Women treated (proportion of those positive for syphilis)‡	889/894	100·0% (99·7 to 100·0)	534/991	43·2% (2·6 to 83·2)	56·8% (12·8 to 99·0)	0·0028
**Secondary outcomes**						
Women screened for anaemia at their first clinic visit	8666/18 357	50·0% (22·3 to 75·8)	8097/17 679	57·0% (27·7 to 70·5)	−7·0% (−24·9 to 30·8)	0·72
Women screened for proteinuria at their first clinic visit	2171/18 355	0·8% (0·3 to 7·2)	1458/17 677	0·1% (0·0 to 1·8)	0·7% (−0·2 to 6·3)	0·22
Women screened for HIV at their first clinic visit	15 422/18 320	86·5% (78·1 to 90·5)	14 189/17 678	81·1% (68·4 to 84·8)	5·4% (−2·4 to 15·9)	0·10

* Wilcoxon rank sum test.

† The clinic is the unit of analysis.

‡ For three clinics in the control group in the Democratic Republic of the Congo, the proportion of women screened positive for syphilis who were treated at the first visit could not be calculated because the clinic had no women who were positive for syphilis.

In the intervention clinics, a median of 100% (IQR 99·7–100·0) of women who were screened for syphilis and tested seropositive during their first visit were treated (table 2). However, in control clinics the proportion was 43·2% (2·6–83·2). The absolute difference was 56·8% (95% CI 12·8–99·0; p=0·0028). 12 (92%) of the 13 intervention clinics treated more than 95% of seropositive women. Of the 13 control clinics, two (15%) treated more than 95% of women; eight (62%) treated 0–87%, and three clinics had no proportion calculated because none of the women at that clinic were seropositive during the follow-up period (figure 2B). In the Lusaka clinics, 868 (6·3%) of 13 829 women in the intervention clinics and 988 (6·8%) of 14 504 in the control clinics were seropositive for syphilis; in Kinshasa, 26 (0·6%) of 4485 women in the intervention clinics and six (0·2%) of 2529 in the control clinics were seropositive. The intervention effect on the treatment outcome was different between Kinshasa (100·0%) and Lusaka (44·6%; appendix).

**Figure 3:**
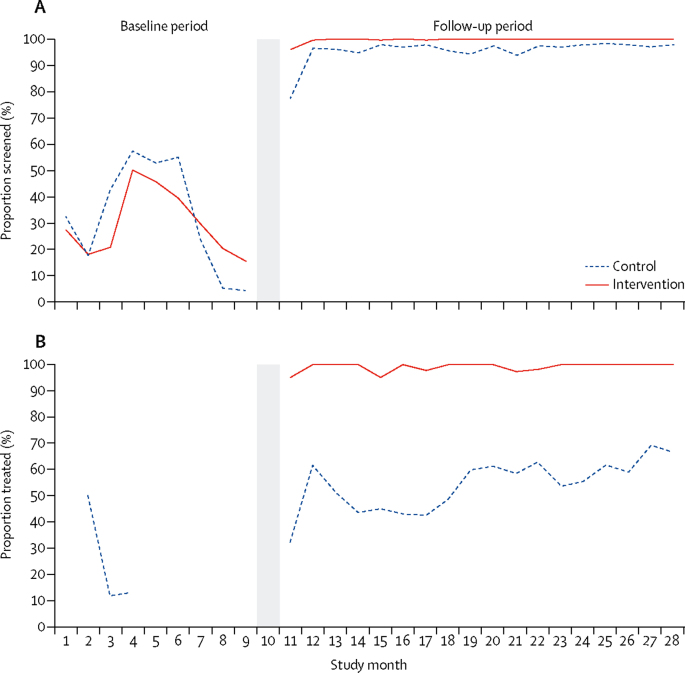
Syphilis screening and treatment by study month Proportions of women screened of total number of pregnant women attending clinic (A) and proportions of women treated of total number of women who tested seropositive for syphilis (B) over the baseline and follow-up periods. The grey area indicates when the intervention was implemented, separating the baseline and post-intervention periods.

For both outcomes, increases were observed after the 9-month baseline period, at the time when distribution of supplies to both trial groups was initiated (figure 3). Proportions of seropositive women treated in the control group increased slightly over time.

Regarding the secondary outcomes, no statistically or clinically significant differences were recorded in the proportions of women screened for anaemia, HIV, and proteinuria between the intervention and control clinics (table 2).

We followed up all seropositive women that were not treated at their first visit, irrespective of trial group. In all 26 clinics, 462 women who tested seropositive at screening were not treated at their first visit; 383 (83%) of 462 were followed up during pregnancy and 380 (99%) of these 383 women received at least one dose of benzathine benzylpenicillin.

No adverse reactions were reported among the 1422 women who received benzathine benzylpenicillin treatment at their first visit at all participating clinics. Only one seropositive woman received erythromycin treatment.

## Discussion

In this cluster randomised controlled trial, we showed that in antenatal care clinics in Kinshasa and Lusaka, a behavioural intervention, including the use of opinion leaders, academic detailing visits, reminders, audit and feedback, and supportive supervision, combined with the provision of kits with supplies, resulted in almost 100% of pregnant women being screened during antenatal care, and 100% of seropositive women being treated at the first visit. Our results showed that for every 1000 pregnant women attending antenatal care in clinics applying our intervention, 61 more women were screened for syphilis than in similar clinics receiving supplies only. Moreover, for every 1000 pregnant women found seropositive for syphilis at screening, 568 more women were treated during their first antenatal care visit in the clinics receiving the multifaceted intervention than in the clinics receiving supplies only. No effects were observed on other antenatal care practices not targeted by the intervention, including screening for anaemia, HIV, and proteinuria.

This trial had several strengths. The experimental design was rigorous and attained similar groups by using covariate-constrained randomisation. Careful training and monitoring of data collectors, who were independent from the antenatal care clinics, resulted in unbiased high-quality data acquisition with no missing data on primary outcomes. The selected behavioural strategies using the conceptual framework of diffusion theory^15^ were documented as effective in changing behaviour^8^ and were tailored according to formative research.^16^ Furthermore, these strategies were successfully integrated into routine care in representative clinics of the existing health systems in both countries, suggesting that the observed effects might be similar in antenatal care programmes incorporating these components.

The study had some limitations. The main aim of the study was to improve syphilis screening and treatment at the first antenatal care visit; however, for ethical reasons, all women not treated at their first visit in either the control or intervention group were followed up to ensure as far as is possible that all seropositive women were treated before delivery of their child. This decision prevented the study from assessing the effect of the intervention on treatment during the whole pregnancy period or on perinatal outcomes at delivery. Although the study enrolled more than 18 000 pregnant women, the very small numbers of women who were seropositive in the Kinshasa clinics were unanticipated when we selected the clinics. Of the nearly 2000 women screened seropositive for syphilis in both cities, only 32 were from clinics in Kinshasa. These low figures prevented the study team from calculating infection rates in three control clinics and yielded a very small cluster size for the remaining clinics in Kinshasa. Nevertheless, restricting the analysis to clinics in Zambia showed a similar and statistically significant positive effect of the intervention on the treatment outcome. Additionally, the behavioural intervention was implemented as a package; thus, the contribution of each specific behavioural component cannot be disentangled from the overall intervention effect. Furthermore, in the context of a pragmatic implementation trial, point-of-care tests were used in routine field conditions and quality assurance tests were not done.

The intervention combining the provision of supplies with behavioural components was associated with screening of practically 100% of pregnant women. However, providing only supplies also attained high coverage, with screening rates of 94%. Whether the difference in screening coverage between the two strategies is important from a public health perspective is a matter of discussion. The sole provision of supplies accompanied with minimal training seems to be enough to achieve high proportions of women screened, and the difference that can be attained by adding the behavioural strategies might not be worth the complexity and costs of implementing and maintaining such interventions. This finding is in agreement with a recently published implementation research antenatal care trial in Mozambique,^6^ in which the provision of kits with supplies led to syphilis screening of 95·5% of patients. Using HIV-syphilis dual tests is another promising approach to increase syphilis screening.^21^ However, in our study, the provision of supplies alone resulted in syphilis treatment on the day of testing in less than 50% of seropositive women. The addition of a behavioural component increased treatment up to 100%, suggesting that it changed some entrenched attitudes against immediate treatment on the same day, such as unawareness that one dose of benzathine benzylpenicillin is enough to prevent mother-to-child syphilis transmission and concerns about potential adverse reactions to benzathine benzylpenicillin.^16^ The effects on proportions of patients treated also agree with the antenatal care trial in Mozambique.^6^ In that study, the provision of kits with supplies for syphilis rapid screening and treatment increased the proportions treated from 60·8% to 86·2%, a smaller effect than that observed for screening. Similarly, one cluster trial assessing point-of-care syphilis screening with test results on the same day showed no differences in proportions of patients treated compared with conventional laboratory strategies.^22^ To our knowledge, only one cluster trial, done in Ulaanbaatar, Mongolia, assessing a point-of-care screening strategy, showed treatment in more than 95% of participants.^23^ Notably, in that study, the proportions of patients treated in clinics using conventional laboratory screening methods were almost 90%, which is higher than the proportions at baseline or in control groups in the studies conducted in sub-Saharan Africa.^6^, ^22^

The absence of observed effects on other antenatal care screening practices, such as screening for anaemia, proteinuria, or HIV, suggests that the effects of our syphilis intervention did not affect practices not specifically targeted by the intervention.

In summary, a behavioural intervention, together with the provision of supplies, can achieve syphilis screening and treatment in more than 95% of women, which is the suggested coverage proposed by WHO towards the elimination of mother-to-child transmission of syphilis.^4^, ^5^ The sole provision of supplies is sufficient to reach such levels of screening coverage but is not sufficient to ensure high levels of treatment.

In our opinion, the results of the trial are generalisable to antenatal care clinics in large urban settings of sub-Saharan countries. Further research is needed to assess the cost-effectiveness of this strategy and its generalisability to other geographic areas and cultures as well as to other care practices. Attrition between diagnosis and appropriate care is a consistent challenge in health care, including major infectious disease programmes such as HIV and tuberculosis.^24^, ^25^ Similar methods have proven effective in changing provider behaviour for other conditions.^8^, ^26^, ^27^

Further research could establish whether the behavioural intervention could be simplified without decreasing its effect.

## Data sharing

De-identified individual participant data (including data dictionaries) and data collected during the trial that underlie the results reported here and the study protocol will be available online for 5 years from publication of this Article. Anyone who wishes can access the data for any purpose without restriction.
