# Supplementary material for: A multifaceted intervention to improve syphilis screening and treatment in pregnant women in Kinshasa, Democratic Republic of the Congo and in Lusaka, Zambia: a cluster randomised controlled trial
Source: Lancet Glob Health. 2019 Mar 22;7(5):e655–63. doi: 10.1016/S2214-109X(19)30075-0 (PMC6465956; doi:10.1016/S2214-109X(19)30075-0)
Supplement: Supplementary appendix [file mmc1.pdf]

THE LANCET
Global Health

Supplementary appendix

This appendix formed part of the original submission and has been peer reviewed. We post it as supplied by the authors.

Supplement to: Althabe F, Chomba E, Tshefu AK, et al. A multifaceted intervention to improve syphilis screening and treatment in pregnant women in Kinshasa, Democratic Republic of the Congo and in Lusaka, Zambia: a cluster randomised controlled trial. *Lancet Glob Health* 2019; published online March 22. http://dx.doi.org/10.1016/S2214-109X(19)30075-0.

A multifaceted intervention to improve syphilis screening and treatment in pregnant women in Kinshasa, Democratic Republic of the Congo and in Lusaka, Zambia: a cluster randomized controlled trial.

Supplementary Appendixes

Appendix A: Institutional reviews boards (IRBs) that approved the protocol

1. Tulane University Institutional Review Board, New Orleans, United States
2. Ethics Committee of the Ecole de Santé Publique, Université de Kinshasa, Republique Democratique du Congo
3. Ethics Committee of the University of Zambia, Lusaka, Zambia
4. Ethics Committee of the Centro de Educación Médica e Investigaciones Clínicas “Norberto Quirno”, Buenos Aires, Argentina.

Appendix B

Intervention description (following TIDieR guide)

<table>
<tr><td>Brief name:</td></tr>
<tr><td>1.¨Supplies+Plus¨: a behavioral multifaceted intervention involving supplies kits plus a behavioral/implementation component</td></tr>
<tr><td>Why:</td></tr>
<tr><td>2. Implementation research suggests that additional interventions are needed to change the behaviors of health providers and that stand-alone supplies provision might be not enough to improve levels of care. Behavioral interventions based on the stages of change and organizational change theories (diffusion of innovation theory) [1] tailored by a previous formative research has been proven effective [2, 3, 4]</td></tr>
<tr><td>What:</td></tr>
<tr><td>Intervention clinics received the “Supplies plus” package intervention that consisted of: selection of facilitators, academic detailing visits with ANC providers, use of reminders, audit and feedback, supportive supervision, and kits with supplies for syphilis testing and treatment, as well as training regarding their use and stock resources management.
3.Materials
The intervention included the delivery and organization of supplies for syphilis screening and treatment of pregnant women during the ANC visit. Supplies were packaged in 3 different kits: diagnostic, treatment and anaphylaxis kits. The contents of each kit are detailed below:
a. Diagnostic Kit:
This kit contained all supplies needed for on-site diagnosis of syphilis:
✓ Syphilis rapid test (Alere in DRC and SD Bioline in Zambia)
✓ Lancet
✓ Capillary tube
✓ Instructions of use and for immediate treatment if positive
✓ Buffer solution for test use.
✓ Alcohol pad
✓ Gloves
b. Treatment Kit:
The contents of this kit were aimed for on the spot treatment and consisted in:
✓ 1 vial of benzathine penicillin 2.4 MU
✓ Erythromycin capsules
✓ 1 Syringe (5 or 10 ml)
✓ 1 Needle for IM injection
✓ Instructions of use
✓ Information on side-effects
✓ Alcohol pad
✓ Gloves
c. Anaphylaxis Kit:
This kit contained all the supplies needed to treat anaphylaxis, according to local practice guidelines:
✓ 1 vial of hydrocortisone (doses)
✓ 1 vial of adrenaline (doses)
✓ Syringes
✓ Needles
✓ Instructions of use
✓ Information on side-effects</td></tr>
</table>

- ✓ Alcohol pad
- ✓ Gloves

In Lusaka the diagnostic, treatment and anaphylaxis supplies were packaged in fishing boxes, using the different sections for each type of resource. DRC sites used small and big plastic boxes to place and organize the resources with the corresponding labels. Both sites placed the kits with an identificatory sticker of the study in the ANC office where women were provided care. **Stock cards** were filled by facilitators on a daily basis and they were trained to recognize the minimum stock levels to prevent supply stock out.

Reminders, created by the selected facilitators and the rest of ANC health providers, consisted on simple messages and figures to remind prenatal health providers on how to conduct syphilis screening procedures and how to provide treatment for those women found positive at the first ANC visit. Reminders had different formats (posters, aprons, stickers, t-shirts) and were displayed in selected strategic places at the clinics (waiting rooms and offices) and were updated as needed by the facilitators and staff. Reminder´s contents were based on syphilis screening and treatment during the first antenatal visit, and on treatment management of adverse effects of positive women.

On a monthly basis, a simple **monitoring report** with the screening and treatment rates of positive women for syphilis at the first antenatal visit was given to the facilitators by the site intervention coordinator to be used in feedback sessions to prenatal health providers.

4. Procedures

A site intervention coordinator was selected and trained in each site by the study intervention coordinators in a 2 days' workshop. After that, he/she contacted authorities at the intervention clinics in order to summarize the study and organize the selection of facilitators and the training meetings (**Train the trainers**). One to three ANC providers were identified as **facilitators** by their peers ((i.e. those identified as knowledgeable, humane, and liking to share knowledge) at each intervention clinic with the use of a previously validated sociometrical questionnaire [11]. Selected facilitators were trained by the site intervention coordinator in a 2-day workshop in each country, focusing on how to implement, disseminate and monitor the congenital syphilis prevention intervention at the first ANC visit how to screen syphilis during pregnancy at the first ANC visit, training on the use of point-of-care tests, how to treat seropositive pregnant women at the first ANC visit and counsel them and their partners, following the local guidelines. Also, facilitators were trained on how to monitor the supplies stock (storage, packaging and stock management). At the clinics, the facilitators, with the support of the intervention site coordination, replicated the training to the other ANC providers. To facilitate the dissemination, the facilitators conducted **academic detailing visits** to each ANC providers to detect problems regarding testing and treatment for syphilis and determine the need of re training through the intervention period.

The intervention site coordinator conducted monthly **supportive supervision visits** at each intervention clinic. Those activities included provision of support and feedback to the facilitators to ensure the implementation and dissemination of the intervention among ANC providers, problems solving, and sharing and discussing the monthly report with the clinics syphilis screening and treatment rates. Also, they remained the facilitators about the importance of partner testing and treatment following the local guidelines. Supervisory visits were organized in advanced using a specific monthly visit checklist. After the supportive supervision visits, each facilitator distributed the report with the testing and treatment rates among ANC providers and gave them **feedback** to improve the implementation of intervention.

Who provided:

5. The intervention was instrumented by peer nominated facilitators which were current antenatal care health providers (midwifes or nurses) with the following skills: Good communicators (meaning that they enjoy and are willing to share knowledge, never seem too busy to be helpful, and offer clear and practical information) Updated (current and up to date, and demonstrate a high level of expertise) "Human" (when dealing with his/her colleagues and patients, caring providers, never talking down to their colleagues). Specific training on screening,

treatment and counselling was provided during a 2 days' workshop that also included basis of stock management. This was a voluntary unpaid position as part of their normal role.<br>In DRC, initially at the workshop 18 facilitators were trained (2-3 facilitators per clinic) and in Zambia, 12 facilitators were trained (2-3 - facilitators per clinic).
**How:**
6. The multifaceted and educational intervention was focused on the ANC providers and was delivered using face to face train the trainers' methodology. The intervention study coordinators trained the site intervention coordinator in each country in a local workshop. Afterwards, the site intervention coordinator trained all the selected facilitators in a group training workshop in each country. Facilitators at the clinics, with the support of the intervention site coordination, replicated the training to the other ANC providers to enhance skills on screening, treatment and counsel on syphilis during pregnancy. Additionally, facilitators developed themselves during the workshop reminders on syphilis testing to be placed in the waiting rooms and ANC offices. The local coordination office administrated the supplies and delivered them to each intervention clinic. There facilitators packed the supplies in special boxes or cabinets to make them available for use as well as verifying that there was enough stock for testing and treatment. Finally, each pregnant woman at the first ANC visit was offered individually to be screened for syphilis and subsequently treated if needed.<br>ANC providers at all participating clinics were trained to administer benzathine penicillin 2.4 MIU one dose intramuscular, to every woman with a positive RST result at the first ANC visit in order to prevent congenital syphilis. Women who referred being allergic to penicillin were treated with erythromycin 500 mg orally.
**Where:**
7. Intervention was delivered at 13 intervention antenatal care (ANC) clinics in Kinshasa (DRC) and in Lusaka (Zambia) from the public, private and confessional sector in each country. Clinics served a mean of 300 new pregnant women per year, had antenatal care providers qualified to perform screening tests and administer injectable treatments if needed, and did not have any active quality improvement programs for syphilis detection and management.
**When and how much:**
8. The intervention lasted 18 months, where after the initial training, ANC providers were supported by<br>The facilitators on the continuity of the screening and treatment strategy at the first ANC visit for all pregnant women. Provision of feedback regarding the performance, ensuring that the gained skills persisted, and stock management were routinely replenished.
**Tailoring:**
9. N/A
**Modifications:**
10. A re-training session was held only in the Lusaka clinics (due to the availability of sample reading pictures) primarily to reinforce ANC health providers on the reading of syphilis results on the syphilis test. In addition, knowledge and skills on the correct technique for blood draw were revised and practiced. Subsequently, replication of the retraining to health care workers by facilitators was done in all five Intervention clinics at Lusaka. However, as we did not have the same evaluated set for Kinshasa, we decided to re train also the eight intervention participating clinics in that city.
**How well:**
11. Overall the compliance with the intervention was very good as shown by the intervention process measures. During the presentation of the intervention, the median rate for proportions (P25-P75) number of antenatal care staff that attended to the meeting among all health providers by clinic was 86.0% (range 67·5-91·0) in Zambia and 100% (91.0-100.0) in DRC, while 87% (67.5-91.0) and 100.0 (100.0-100.0) of the health providers consented to participate in the intervention at each country respectively. Regarding the facilitators workshop, in Zambia

100% of the nominated health providers that accepted to participate as facilitators attended, in contrast with DRC, were 36·5 (29·0-85·0) participated of the activity. Lastly, in both sites 100.0% (100.0-100.0) of antenatal health providers at the clinics attended to the workshop by the facilitators.

Regarding the supplies component, both sites reported a 100% adherence in relation to the packing of supplies in boxes, meaning the availability of the packing boxes with supplies in the ANC office and to compliance to the minimum stock level required for diagnostic and treatment test. Anaphylaxis test showed a slight variation in DRC with a median of 94·0 (94·0-98·5).The behavioral component, was assessed by the use of reminders as specified reporting an adherence rate of 100% in Zambia and 88% in DRC; facilitators performing at least one academic visit to each health provider accomplishing 100% in both sites; monitoring and feedback with the performance and distribution of the monthly reports to health providers reporting rates of 87·0 (76·5-93·5) in Zambia and 94·0 (94·0-100·0) in DRC; and the expected supportive supervision visits reaching 94·0 (94·0-94·0) in Zambia and 103·0 (100·0-106·0) in DRC.

Citations:

1. Remme J, Adam T, Becerra-Posada F, et al. Defining research to improve health systems. *PLoS Medicine* 2010; 7(11):e1001000

2. Althabe F, Buekens P, Bergel E, Belizan JM, Campbell MK, Moss N, et al. A behavioral intervention to improve obstetrical care. N Engl J Med. 2008 May;358(18):1929–40

3. Buekens P, Cafferata ML, Alger J, et al. Congenital transmission of Trypanosoma cruzi in Argentina, Honduras, and Mexico: study protocol. *Reprod Health* 2013; 10:55

4. Althabe F, Belizán JM, Mazzoni A, et al. Antenatal corticosteroids trial in preterm births to increase neonatal survival in developing countries: study protocol. *Reprod Health* 2012; 9:22.

5. Chavane L, Merialdi M, Betran A, et al. A demonstration project for the implementation of the WHO antenatal care model in Mozambique: A cluster randomized controlled trial: Study protocol. *BMC Health Serv Res* 2014; 14:228

6. EM. R. Diffusion of Innovations. 3rd ed New York, NY Free Press. 1983

2. Althabe F, Buekens P, Bergel E, Belizan JM, Campbell MK, Moss N, et al. A behavioral intervention to improve obstetrical care. N Engl J Med. 2008 May;358(18):1929–40.

7. Althabe F, Belizán JM, McClure EM, Hemingway-Foday J, Berrueta M, Mazzoni A, et al. A population-based, multifaceted strategy to implement antenatal corticosteroid treatment versus standard care for the reduction of neonatal mortality due to preterm birth in low-income and middle-income countries: The ACT cluster-randomised trial. Lancet. 2015;385(9968).

8. Althabe F, Alemán A, Berrueta M, Morello P, Gibbons L, Colomar M, et al. A Multifaceted Strategy to Implement Brief Smoking Cessation Counseling During Antenatal Care in Argentina and Uruguay: A Cluster Randomized Trial. Nicotine Tob Res. 2016;18(5).

9. Lowe JB, Balanda KP, Stanton WR, Del Mar C, O´Connor V. Dissemination of an Efficacious Antenatal Smoking Cessation Program in Public Hospitals in Australia: A Randomized Controlled Trial. *Health Educ Behav* 2002; 29; 608

10. Deci EL, Ryan RM. The support of autonomy and the control of behavior. *J Pers Soc Psychol* 1987; 53:1024–37.

11. Hiss RG, MacDonald R DW. Identification of physician educational influences in small community hospital. Proc 17th Annu Conf Res Med Educ Washington, DC Assocation Am Med Coll 1978283-8.

Appendix C

Table C.1: Intervention Process Measures (figures are median rates in percentages)

	Kinshasa (n=8)	Lusaka (n=5)	GLOBAL (n=13)
**Measure of intervention Launch**			
**Presentation of the intervention and selection of facilitators**			
Percentage of antenatal care staff attended to the meeting by clinic	100·0 (91·0-100·0)	86·0 (67·5-91·0)	94·0 (86·0-100·0)
Percentage of antenatal care staff consented to participate in the intervention study	100·0 (100·0-100·0)	87·0 (67·5-91·0)	100·0 (80·5-100·0)
**Facilitator Workshop**			
Percentage of nominated health providers that accepted to participate as facilitators	36·5 (29·0-85·0)	100·0 (100·0-100·0)	100·0 (100·0-100·0)
**Health Providers Workshop by facilitators**			
Percentage of antenatal health providers at the clinic that attended to the workshop	100·0 (100·0-100·0)	100·0 (100·0-100·0)	100·0 (100·0-100·0)
**Supplies Component**			
**Packaging of supplies in boxes**			
Availability of packaging boxes with supplies in ANC office	100·0 (100·0-100·0)	100·0 (100·0-100·0)	100·0 (100·0-100·0)
**Minimum stock required available for:**			
Diagnosis test	100·0 (100·0-100·0)	100·0 (100·0-100·0)	100·0 (100·0-100·0)
Treatment	100·0 (100·0-100·0)	100·0 (100·0-100·0)	100·0 (100·0-100·0)
Anaphylaxis	94·0 (94·0-98·5)	100·0 (100·0-100·0)	100·0 (94·0-100·0)
**Implementation – Behavioural Component**			
**Reminders**			
Use of reminders as specified	88·0 (88·0-88·0)	100·0 (100·0-100·0)	88·0 (88·0-100·0)
**Facilitators**			
At least one academic visit to each health provider	100·0 (100·0-100·0)	100·0 (100·0-100·0)	100·0 (100·0-100·0)
**Monitoring and feedback**			
Monthly reports to health providers performed and distributed	94·0 (94·0-100·0)	87·0 (76·5-93·5)	94·0 (87·0-100·0)
**Supportive supervision**			
Percentage of supportive supervision visits over expected	103·0 (100·0-106·0)	94·0 (94·0-94·0)	100·0 (94·0-106·0)

Table C.2: Characteristics of women at follow-up period considering the clinic as the unit of analysis

	Supplies Plus Group (N =13 clinics) (N=18357 women)	Control Group (N =13 clinics) (N=17679 women)
	Median (IQR)	Median (IQR)
**Women's characteristics***		
Maternal age <20	16·4 (14·5-18·4)	17·4 (14·6-22·6)
Incomplete primary school or less	13·1 (10·7-21·4)	22·7 (11·3-25·6)
Married or with partner	91·3 (89·2-92·0)	88·6 (85·8-91·4)
No previous pregnancies	23·1 (21·2-24·9)	24·6 (23·7-27·2)
Previous abortion	26·5 (11·6-29·3)	25·1 (9·8-27·4)
Previous preterm birth	1·6 (1·0-1·9)	2·3 (1·1-2·9)
Previous low birth weight baby	1·6 (0·5-4·7)	3·1 (1·5-6·3)
Previous babies with congenital anomalies	0·2 (0·1-0·3)	0·4 (0·0-0·7)
Previous syphilis infection	0·0 (0·0-1·2)	0·4 (0·0-1·3)
Gestational age at first visit <= 20 weeks	46·9 (38·2-58·2)	44·9 (39·1-51·2)

*The clinic is the unit of analysis

Table C.3: Effect of the intervention in Primary and Secondary outcomes, Kinshasa

	Supplies Plus Group		Control Group		Difference of the medians (95%CI)	P-value£
	(N = 4527 women)	(N =8 clinics)	(N = 3034 women)	(N =8 clinics)		
	n/N	Median Rate*	n/N	Median Rate*		
**Primary Outcomes**						
Women screened for syphilis	4485/4527	99·2	2529/3034	85·9	13·3 (5·2 ; 29·3)	0·0019
Women screened positive for syphilis and treated **	25/26	100·0	2/6	0·0	100·0 (0·0 ; 100·0)	0·0584
**Secondary Outcomes**						
Women screened for anemia at their first ANC visit	1898/4527	42·3	1283/3034	46·9	-4·6 (-36·5 ; 45·4)	0·7132
Women screened for proteinuria at their first ANC visit	925/4526	3·1	237/3033	0·0	3·1 (0·0 ; 24·8)	0·1389
Women screened for HIV at their first ANC visit	3637/4527	85·1	2019/3034	72·1	13·0 (-10·5 ; 28·6)	0·2701

*The unit of analysis is the clinic
** For 3 clinics at the Control Group the proportion of women screened positive for syphilis who are treated at the first visit could not be calculated because the clinic has no woman positive for syphilis
£Wilcoxon rank sum test

Table C.4: Effect of the intervention in Primary and Secondary outcomes, Lusaka

	Supplies Plus Group		Control Group		Difference of the medians (95%CI)	P-value£
	(N = 13830 women)	(N =5 clinics)	(N = 14645 women)	(N =5 clinics)		
	n/N	Median Rate*	n/N	Median Rate*		
**Primary Outcomes**						
Women screened for syphilis	13829/13830	100·0	14507/14645	98·9	1·1 (0·0 ; 1·5)	0·0449
Women screened positive for syphilis and treated	864/868	99·7	532/985	55·1	44·6 (12·5 ; 89·2)	0·0119
**Secondary Outcomes**						
Women screened for anemia at their first ANC visit	6768/13830	61·8	6814/14645	59·6	2·2 (-51·2 ; 52·1)	1·0000
Women screened for proteinuria at their first ANC visit	1246/13829	0·8	1221/14644	0·6	0·3 (-87·6 ; 85·7)	1·0000
Women screened for HIV at their first ANC visit	11785/13793	86·6	12170/14644	83·5	3·1 (-5·3 ; 8·7)	0·2101

*The unit of analysis is the clinic
£Wilcoxon rank sum test
